# A Threshold Model for T-Cell Activation in the Era of Checkpoint Blockade Immunotherapy

**DOI:** 10.3389/fimmu.2019.00491

**Published:** 2019-03-18

**Authors:** Kripa Guram, Sangwoo S. Kim, Victoria Wu, P. Dominick Sanders, Sandip Patel, Stephen P. Schoenberger, Ezra E. W. Cohen, Si-Yi Chen, Andrew B. Sharabi

**Affiliations:** ^1^Department of Radiation Medicine and Applied Sciences, San Diego Moores Cancer Center, University of California, San Diego, San Diego, CA, United States; ^2^Moores Comprehensive Cancer Center, University of California, San Diego, San Diego, CA, United States; ^3^Division of Hematology and Oncology, Center for Personalized Cancer Therapy, San Diego Moores Cancer Center, University of California, San Diego, San Diego, CA, United States; ^4^Laboratory of Cellular Immunology, La Jolla Institute for Allergy and Immunology, La Jolla, CA, United States; ^5^Department of Molecular Microbiology and Immunology, Norris Comprehensive Cancer Center, University of Southern California, Los Angeles, CA, United States

**Keywords:** negative regulator, antigen presentation attenuator, threshold model, checkpoint blockade, immunotherapy, PD-1, CTLA-4, T cell exhaustion

## Abstract

Continued discoveries of negative regulators of inflammatory signaling provide detailed molecular insights into peripheral tolerance and anti-tumor immunity. Accumulating evidence indicates that peripheral tolerance is maintained at multiple levels of immune responses by negative regulators of proinflammatory signaling, soluble anti-inflammatory factors, inhibitory surface receptors & ligands, and regulatory cell subsets. This review provides a global overview of these regulatory machineries that work in concert to maintain peripheral tolerance at cellular and host levels, focusing on the direct and indirect regulation of T cells. The recent success of checkpoint blockade immunotherapy (CBI) has initiated a dramatic shift in the paradigm of cancer treatment. Unprecedented responses to CBI have highlighted the central role of T cells in both anti-tumor immunity and peripheral tolerance and underscored the importance of T cell exhaustion in cancer. We discuss the therapeutic implications of modulating the negative regulators of T cell function for tumor immunotherapy with an emphasis on inhibitory surface receptors & ligands—central players in T cell exhaustion and targets of checkpoint blockade immunotherapies. We then introduce a Threshold Model for Immune Activation—the concept that these regulatory mechanisms contribute to defining a set threshold of immunogenic (proinflammatory) signaling required to elicit an anti-tumor or autoimmune response. We demonstrate the value of the Threshold Model in understanding clinical responses and immune related adverse events in the context of peripheral tolerance, tumor immunity, and the era of Checkpoint Blockade Immunotherapy.

## Introduction

**Figure 1:**
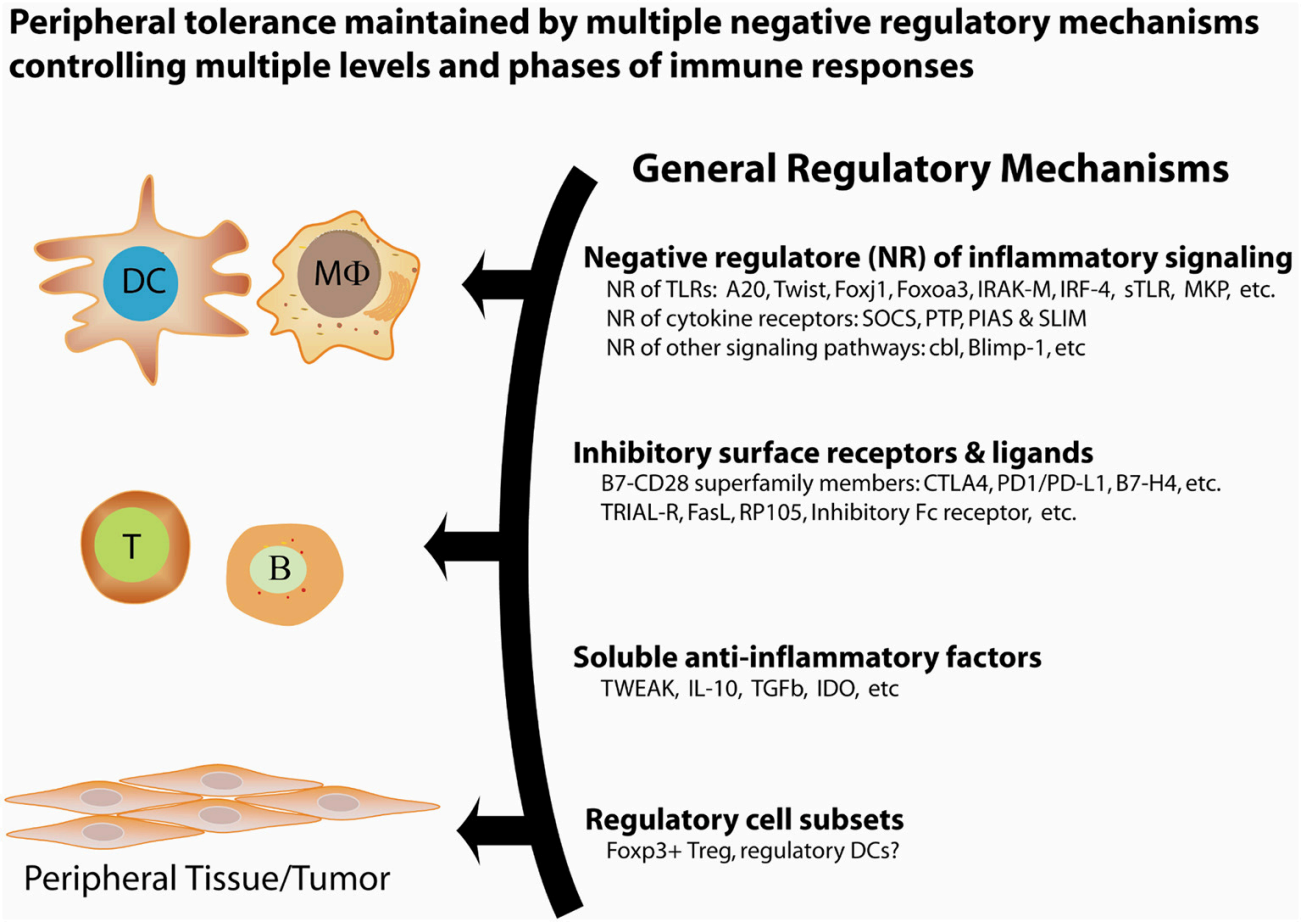
General regulatory mechanisms for the maintenance of peripheral tolerance. Peripheral tolerance is maintained by at least four interrelated, non-redundant regulatory mechanisms that work in concert to negatively regulate multiple levels of immune responses, including antigen presentation, lymphocyte activation and effector function, and peripheral tissues.

Over the past decades, an increasing body of genetic studies on the negative regulation of proinflammatory signaling has demonstrated the essential role of negative regulators of proinflammatory signal transduction pathways in the maintenance of peripheral tolerance. Genetic deficiency in one of these negative regulators often results in severe inflammation and autoimmune pathologies in both mice and humans (Tables 1, 2). These genetic studies have provided insights into the nature of the signals, molecular processes, and regulatory cell subsets for maintaining peripheral tolerance. Peripheral tolerance is maintained at multiple levels of immune responses, including (a) antigen presentation, (b) lymphocyte activation and effector function, and (c) peripheral tissues. Each level of immune responses is regulated synergistically by employing diverse regulatory mechanisms, including negative regulators of proinflammatory signaling, soluble anti-inflammatory factors, inhibitory surface receptors & ligands, and regulatory cell subsets (Figure 1). These interrelated, non-redundant regulatory machineries work in concert to ensure an appropriate innate and adaptive immune response that is sufficient to clear invading pathogens, while preventing toxicity from over activation or pathological autoimmunity.

**Table 1:** Representative negative regulators of TLR and related signal transduction pathways.

	**Negative regulators**	**Example**	**Expression**	**Possible mechanism**	**Phenotype of genetic deficient mice**	**References**
Ubiquitin E3 ligases/SUMO	E3 ubiquitin ligases (hundreds of members)	TRIAD3A	Constitutive	Ubiquitinates TLRs for degradation	NA	(1)
		SOCS1	Inducible	Ubiquitinates Mal for degradation, in addition to inhibiting JAK/STAT	Neonatal lethality, severe inflammation of multiple organs, hypersensitivity to LPS, hy peractivated DCs, MΦ, and T cells	(2)
		A20	Inducible	Ubiquitinates/deubiquitinates RIP and TRAF6 for degradation	Neonatal death, severe inflammation, cachexia and hypersensitivity to LPS	(3–5)
	SUMO (4)	SUMO1	Constitutive	SUMOylate and stabilize IκB	NA (not available)	(6, 7)
Inhibitory isoforms	IRAK isoforms	IRAK-M	Inducible	Prevents dissociation of IRAK-IRAK4 and formation of IRAK-TRAF6	No gross abnormality, hypersensitive to LPS, hyperactivated Mϕ	(8)
	MyD88s		Inducible	Inhibits MyD88	NA	(9)
	ST2		Inducible	Inhibits TLR4 signaling by sequestrating MyD88 and Mal	No obvious abnormalities, fail to develop endotoxin tolerance, reduced production of TH2 cytokines	(10, 11)
	SIGIRR		Constitutive, reduced by stimulation	Binds and inhibits TLR4–IL-1R signaling molecules IRAK and TRAF6	No obvious abnormalities, hypersensitive to LPS, hyperactivated Mϕ and T cells	(12)
	RP105		Constitutive	Inhibits TLR4 binding with microbial products	NA	(13)
	sTLR2/4		Constitutive	Antagonists of TLR2 and 4	NA	(14, 15)
Inhibitory components of signaling complexes	IκB (3)	IκBα	Constitutive	Retains NF-κB in the cytosol	Neonatal death, severe dermatitis and inflammation	(16, 17)
	IKKα		Constitutive	Phosphorylates RelA and c-Rel, resulting in accelerated turnover	Neonatal death, enhanced sensitivity to LPS, hyperactivated Mϕ	(17, 18)
Transcription factors	Fox (>100 members)	Foxj1, FoxOa3, Foxp3	Constitutive, reduced by stimulation Constitutive, reduced by stimulation Constitutive in CD25^+^CD4^+^ T cells	Transcription activator of IκBβ Transcription activator of IκBβ/ IκBξ Transcription repressor of proinflammatory cytokines	Embryonic lethality, chimerization of Rag^−/−^ mice results in severe inflammation, hyperactivated T cells No gross abnormality, multiorgan inflammation, lymphoproliferation Neonatal death, inflamed skin (scurfy), severe inflammation of multiple organs, fatal IPEX syndrome in humans	(19) (20) (21–24)
	Twist 1/2		Inducible	Inhibits NF-κB binding to cytokine promoters	Neonatal death, severe inflammation, cachexia, and hypersensitivity to tnf	(25)
Phosphatases (PTP)	MKP (11 members)	MKP1 MKP5	Inducible Inducible in Mϕ,	Inhibits JNK and p38 pathways Inhibits JNK pathway	No gross abnormality Hypersensitive to lps, hyperactivated mϕ No gross abnormality, hypersensitive to lps, hyperactivated mϕ and T cells	(26, 27) (28)
Other mechanisms	Dok-1/2		Constitutive	Suppresses Erk activation of TLR4 signaling	No gross abnormality, hypersensitive to LPS, hyperactivated Mϕ and T cells	(29) (30)
	β-Arrestin-1/2		Constitutive	Binds and inhibits TRAF6, stabilizes IκBα	Hypersensitive to LPS, hyperactivated Mϕ	(31–33)
	TOLLIP		Constitutive	Suppresses IRAK1	NA	(34)
	NOD2		Constitutive	Inhibits TLR2-drived activation of NF-κB and T_H_1 responses	Inflammatory diseases such as colitis, Crohn's disease in humans	(35, 36)

**Table 2:** Representative negative regulators of cytokine receptor signaling pathways.

**Negative regulator**	**Example**	**Expression**	**Possible mechanism on immune responses**	**Phenotype of genetic deficient mice**	**References**
SOCS (8 members)	SOCS1 SOCS2 SOCS3	Inducible Inducible Inducible	Blocks JAK-Stat interaction and ubiquitinates JAK for degradation Inhibits the signaling of growth hormone and cytokines Selectively inhibits IL-6 receptor subunit gp130-mediated signaling	Neonatal lethality, severe inflammation of multiple organs, hypersensitivity to LPS, hyperactivated DCs, MΦ & T cells Gigantism, hypersensitive to microbial stimuli, hyperactivated DCs, Embryonic lethality due to placental defects, mice with a conditional deletion in MΦ and neutrophils are hyposensitive to LPS	(37–41) (42, 43). (44–46).
PIAS (4 members)	PIAS1	Constitutive	Blocks DNA binding of STATs, sumoylates STATs to inhibit their transcription, blocks the DNA binding of p65 to suppress NF-κB	No gross abnormality, hypersensitivity to LPS, hyperactivated MΦ	(47, 48)
PTP (107 members)	SHP1 SHP2	Constitutive Constitutive	Dephosphorylates cytokine receptor signaling molecules Dephosphorylates cytokine receptor signaling molecules	*Motheaten* (dermatitis) phenotype Embryonic lethality due to severe hematopoietic defects	(49, 50) (51)
SLIM		Constitutive	Ubiquitinates STAT1 and STAT4 for degradation	No gross abnormality, enhanced IFN production by T cells	(52)

Naïve CD8^+^ T cells that encounter antigens during immune challenge (e.g., acute infection) set forth a cell-intrinsic program that drives them to expand and differentiate into cytotoxic effector cells that control and eventually clear the pathogen (53). At peak response, these effector T cells secrete high amounts of cytokines [interferon-γ (IFNγ) and tumor necrosis factor (TNF)] and cytolytic molecules (granzymes and perforin). Subsequently, if the antigenic source has been eliminated, most of these effector T cells undergo apoptosis, and a few survive and become central memory and effector memory T cells (54, 55). While this differentiation process is tightly controlled, changes in the nature, context, and duration of antigen exposure can alter the process and lead to T cell dysfunction, unresponsiveness, and/or death. Observed phenotypic and functional features define T cell dysfunction as exhaustion, tolerance, or anergy, and characterizing these cellular and molecular features can define strategies that can overcome their dysfunction. T cell dysfunction has been well-studied in infections associated with high viral replication, like LCMV clone 13, hepatitis C virus, hepatitis B virus, and HIV, but also in bacterial and parasitic infections and cancer (56–60). Here, we discuss the various states of T cell dysfunction, focusing on the more extensively defined characteristics of tolerance and exhaustion in tumor associated T cells.

In connecting these diverse regulatory mechanisms and providing a global overview of T cell dysfunction, we introduce the Threshold Model for Immune Activation—the concept that an individual's immune system has an inherent threshold of immunogenic signaling required to elicit either an anti-tumor immune response, auto-immune response, or both. This threshold is determined by the interplay of these negative regulators and their stimulatory counterparts, which work together to modulate the functional state of individual immune cells and achieve immune homeostasis. Consideration of the delicate balance of T cell activation and exhaustion/tolerance required for homeostasis is critical as we work toward developing immunotherapies that maximize anti-tumor function while minimizing detrimental immunological pathology. As the decades of research in immunology and cancer biology are finally coming to fruition in the clinic, this model provides a conceptual framework to orient ourselves in the Era of Checkpoint Blockade.

## The Threshold Model for Immune Activation

**Figure 2:**
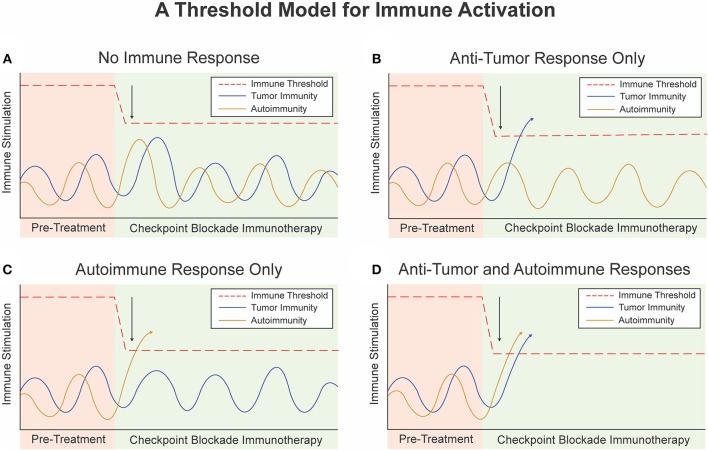
A threshold model for immune activation. **(A)** Checkpoint Blockade Immunotherapy (CBI) lowers the threshold for immune activation but not enough to trigger an immune response. **(B)** CBI lowers the threshold for immune activation to trigger an anti-tumor immune response but not an auto-immune response. **(C)** Baseline immunity to self antigens is higher than immunity to tumor associated antigens. CBI lowers the threshold to induce an auto-immune response but no anti-tumor immune response or immune-related adverse event. **(D)** Single agent or dual agent CBI lowers the threshold for immune activation to trigger an anti-tumor immune response and an auto-immune response or immune-related adverse event.

A vast number of positive and negative regulatory factors modulate the immune system and work in concert to achieve immune homeostasis (61–65). Regulation of the immune system is exceedingly complex, involving the interplay of these diverse regulatory mechanisms at multiple levels. Immune regulation also varies greatly between individuals, and is likely a major factor in differential outcomes and adverse effects seen in patients treated with cancer immunotherapy (66). As described here the Threshold Model provides a straightforward context for understanding immune activation, autoimmunity, and anti-tumor immunity in order to conceptualize the types of responses we have seen in patients treated with CBI:

For an individual patient, a plotted horizontal line represents their immune system's “threshold” of immune activation (Figure 2). The threshold represents the level of immunogenic stimulation required to elicit an immune response, determined by the sum of the negative regulatory mechanisms that work at all levels of the immune system. Stimulation of the immune system to any level below this threshold may still activate individual cells of the immune system, but is insufficient to overcome these regulatory mechanisms and mount an effective systemic immune response.

In a given patient, there are various events that stimulate or inhibit their baseline tumor or host immunogenicity. We can plot the level of immunogenicity over time as sinusoidal “immunogenicity curves” that represent different types of baseline immunogenicity and natural variations in baseline tumor or host immunogenicity due to host and environmental factors (Figure 2). These curves are determined by a multitude of factors, including the patient's genetics, environmental exposures, health, diet, as well as positive and negative feedback mechanisms themselves. Notably, people who are genetically predisposed to autoimmune conditions would have a baseline autoimmune immunogenicity curve with higher peaks than those without this risk factor. For instance, a patient with systemic lupus erythematosus may live many years before developing full blown autoimmunity due to some triggering event which results in crossing of the threshold (67).

At rest, the peak of the immunogenicity curve lies below the threshold of activation, and antigen specific immune responses are not promoted (Figure 2). When the immune system is stimulated beyond the threshold level, such as due to presence of a bacteria, virus, or other exogenous factor an antigen specific immune response occurs (Figure 2). Once the threshold is crossed positive feedback loops and autocrine and paracrine signaling pathways naturally induce rapid expansion and propagation of antigen specific T-cells or B-cells. Negative regulatory mechanisms still play a role in modulating the extent and duration of this response, and eventually bring the immune system back to homeostasis or a new baseline with formation of memory responses.

The first approach to cancer immunotherapy was to enhance positive stimulation of the immune system with bacterial preparations and subsequently cytokines or vaccines to drive anti-tumor immunity (68). This effectively increased the peaks of the baseline tumor immunogenicity curve in attempt to stimulate beyond the threshold. However, due to the powerful negative regulatory mechanisms we will discuss this approach was rarely successful and the threshold for immunity was likely higher than could be reached with these agonist agents alone in most cases.

The approach used with Checkpoint Blockade Immunotherapy (CBI) is to directly inhibit the negative regulatory mechanisms and disable the major brakes on the immune system, including the CTLA-4 or PD-1/PD-L pathways (69, 70). This approach effectively lowers the immune threshold and decreases the amount of stimulation required to elicit an immune response. Dual agent checkpoint blockade would effectively lower the threshold for immune activation even further. We illustrate the possible outcomes of lowering the immune threshold via CBI in Figure 2. The activity of single agent checkpoint blockade alone and the concomitant increase in objective response rates and immune related toxicity with dual agent checkpoint blockade strongly support this threshold model (71). Interestingly, the activation of an anti-tumor immune response may result in development adaptive resistance or emergence of counter-regulatory mechanisms which attempt to subvert this response or effectively raise the threshold back up (72). This could have important implications for maintenance therapy or re-induction of immune responses during re-challenge with CBI after an initial course.

Another important point derived from this model is the concept that more stimulation is not necessarily better for immune responses. In fact excessive stimulation in many cases has a paradoxical response and leads to inhibition or activation induced cell death (73). The immune system must constantly attempt to balance under activation (risk of disseminated infection) and over activation (risk of auto-immunity or cytokine storm). Thus, the threshold model would predict that in the absence of negative regulation (lowest threshold) only a minor amount of positive stimulation would be required to elicit an immune response. Indeed, this is evidenced by the activity of single agent checkpoint blockade alone, where stimulatory factors already present in the tumor microenvironment or lymphoid organs are sufficient to activate potent anti-tumor immunity without the need for exogenous proinflammatory or agonist factors (69, 70). The clinical implication of this being that in the era of checkpoint blockade as we return to testing proimmunogenic agonist therapies less drug may be needed for optimal induction of immune responses in certain circumstances.

In this article we will first highlight the immunologic principles governing negative regulation of immune cells, then more closely examine the role of T cell regulation and T cell exhaustion in cancer. We apply the threshold model to better understand how to overcome these regulatory mechanisms and induce effective anti-tumor immune responses in the setting of checkpoint blockade immunotherapy.

## Characteristics of Immune Tolerance

### Negative Regulation at the Level of Antigen Presenting Cells

**Figure 3:**
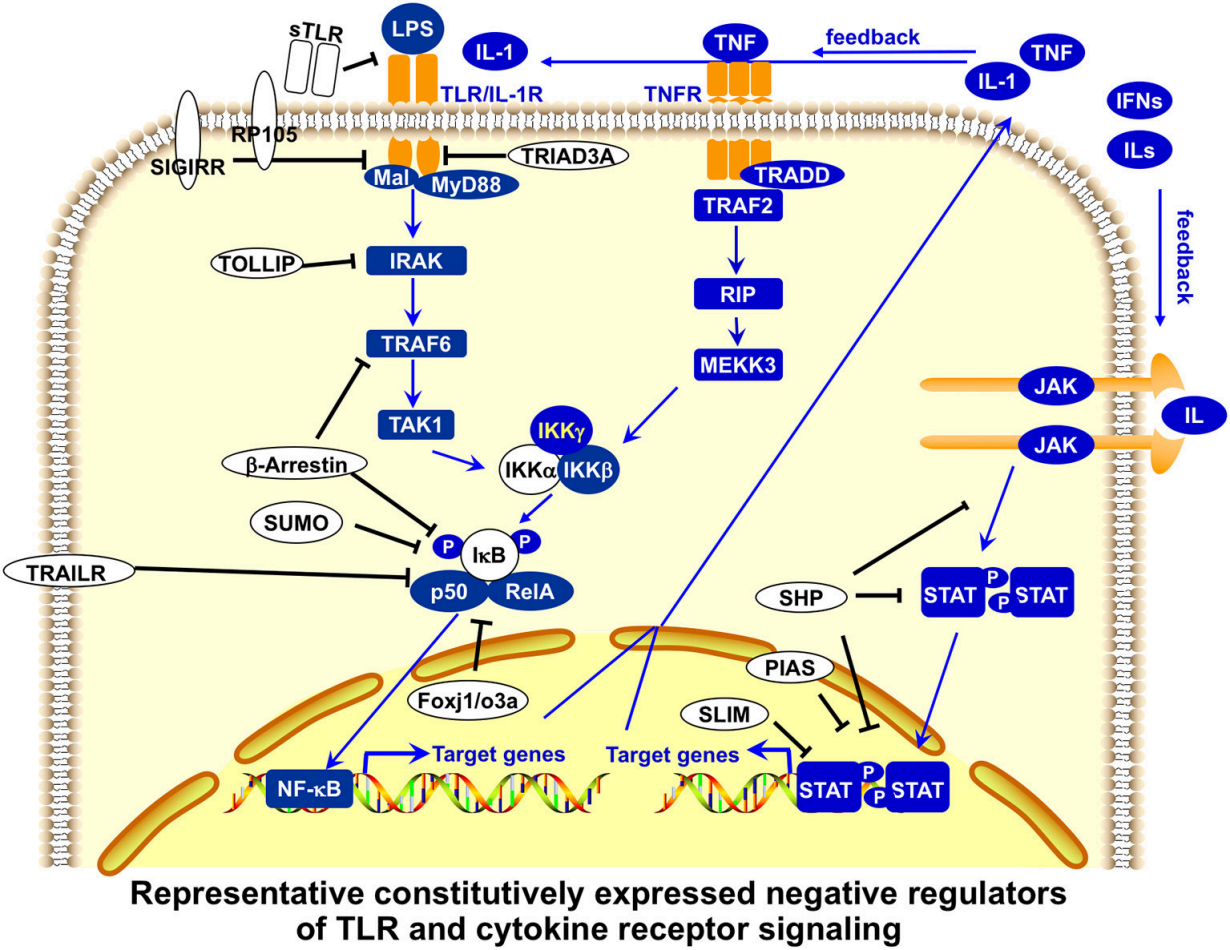
Representative, constitutively expressed negative regulators of TLR and cytokine receptor signaling in APCs. Toll-like receptor (TLR) signaling is regulated by constitutively expressed negative regulators at multiple points. Soluble forms of Toll-like receptors (sTLR) inhibit the binding of membrane-bound TLR to microbial ligands. Membrane-bound SIGIRR (single immunoglobulin interleukin-1-related receptor) binds to TLR4 and IRAK (interleukin-1 receptor-associated kinase), and terminates the downstream TLR signaling pathways, whereas TRAIL-R (tumor-necrosis factor-related apoptosis-inducing ligand receptor) suppresses nuclear factor-κB (NF-κB) activation, perhaps by stabilizing IκB (inhibitor of NF-κB) and protecting it from degradation. Intracellular TRIAD3A ubiquitinates certain TLRs for degradation. TOLLIP (Toll-interacting protein) suppresses IRAK function by inhibiting TLR signaling. NOD2 might inhibit TLR2 signaling by suppressing NF-κB activity. SUMO sumoylates and stabilizes the inhibitor of NF-κB. Transcription factors Foxj1 and Foxoa3 activate expression of the inhibitor of NF-κB. Cytokine receptor signaling is also regulated by constitutively expressed negative regulators at multiple points. SHP proteins dephosphorylate activated JAKs or receptors. PIAS proteins block the binding of STATs and SUMOylate STATs to inhibit their transcriptional activation, whereas STAT-interacting LIM protein (SLIM) ubiquitinates STAT1 and STAT4 for degradation. Many of these negative regulators also play important roles in regulating T-cell activation and function (65).

**Figure 4:**
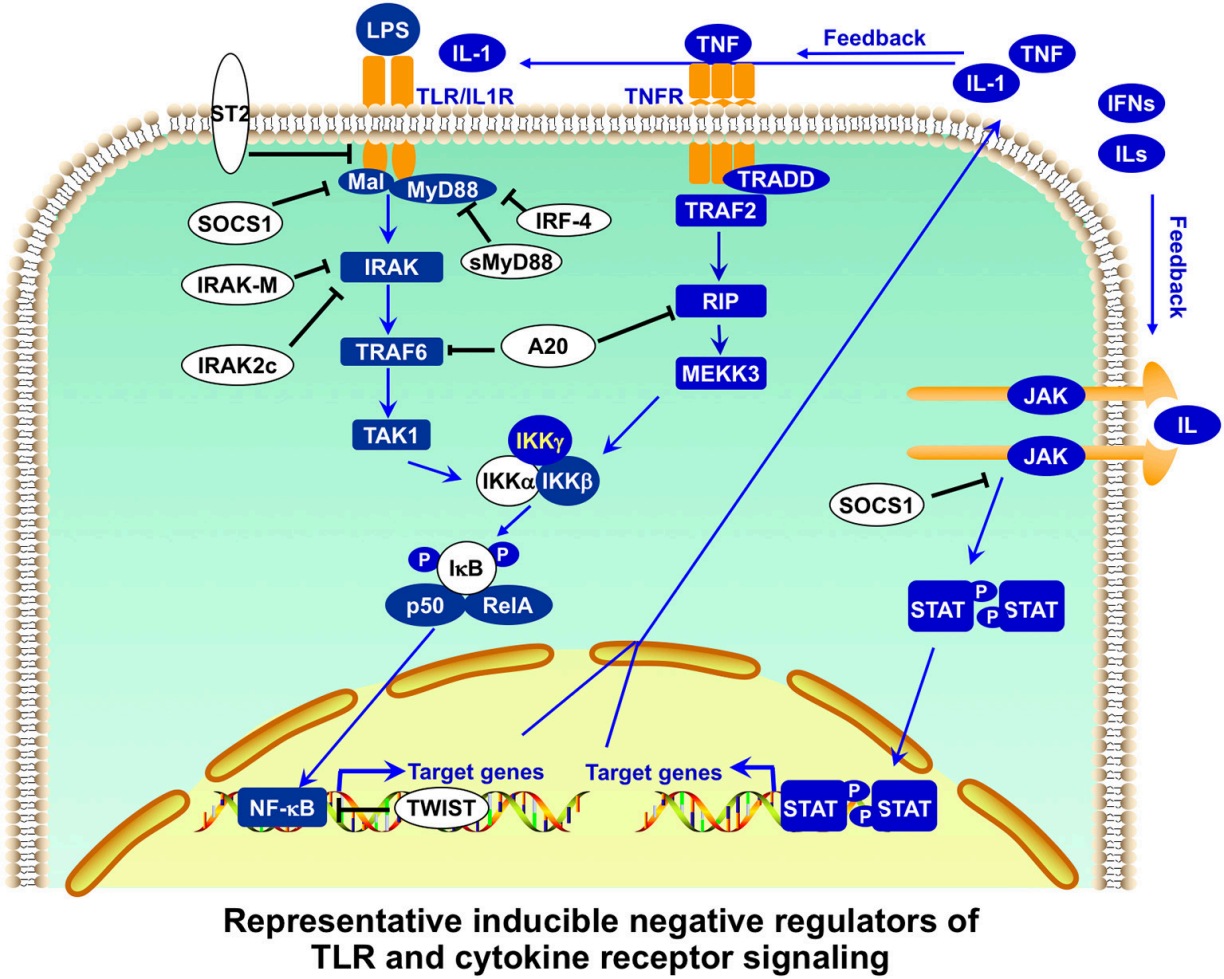
Representative inducible negative regulators of TLR and cytokine receptor signaling in APCs. Toll-like receptor (TLR) signaling is further regulated by inducible negative regulators in a feedback manner. Membrane-bound ST2 interacts with MyD88 and Mal, and sequesters MyD88-dependent nuclear factor-κB (NF-κB) from activation. MyD88s (the short form of MyD88) antagonizes MyD88 functions. Intracellular IRAKM (interleukin-1 (IL-1) receptor-associated kinase M) inhibits the dissociation of IRAK-IRAK4 and subsequent formation of IRAK-TRAF6. SOCS1 promotes the ubiquitination of Mal for degradation. A20 has dual functions of ubiquitination and deubiquitination of RIP and TRAF6 (tumor-necrosis factor-receptor-associated factor 6) for their degradation, inhibiting both TLR and TNFR signaling. Transcription repressor Twist-2 inhibits the transcription of NF-κB-targeted genes. Cytokine receptor signaling is also regulated by inducible negative regulators. SOCS1, in addition to regulating TLR signaling, inhibits JAK activity as a pseudosubstrate or promotes the ubiquitination and subsequent degradation of JAK. Many of these negative regulators also play important roles in regulating T-cell activation and function (65).

The first level of regulation of peripheral T cell tolerance involves the control of antigen presentation. Antigen presentation initiates T cell activation, and antigen-presenting cells (APCs), such as dendritic cells (DCs) and macrophages (MΦ), play a critical role in stimulating immune responses as well as maintaining peripheral tolerance (74, 75). Innate immunity activated by Toll-like receptor (TLR) signaling is crucial in the detection of invading pathogens and the activation of adaptive immunity (76). Members of the TLR family detect conservative microbial molecules, including lipopolysaccharide (LPS), bacterial lipoproteins, flagellin, unmethylated CpG DNA, and viral RNA (61, 63). After ligand binding, TLRs dimerize and undergo the conformational change required for recruitment of downstream signaling molecules, activating MyD88-dependent and MyD88-independent pathways. The subsequent activation of nuclear factor-κB (NF-κB) and mitogen-activated protein (MAP) kinases leads to the expression of a large number of proinflammatory molecules, such as costimulatory molecules and cytokines, for the induction of adaptive immunity (61, 63). The stimulatory potency of TLR signaling in the activation of innate and adaptive immunity is reflected in the complicated negative regulation of TLR signaling at multiple points (Figures 3, 4). An excellent recent review by Liew et al. (65) describes many of these negative regulators, including intracellular IRAK-M, MyD88s, PI3K, TOLLIP, A20, TRIAD3A, and NOD2, soluble TLR2/4, membrane-bound SIGIRR, and ST2.

Much of the study on innate and adaptive immunity has been focused on the identification and characterization of proinflammatory signal receptors and regulators (61, 63). However, during the last two decades, an increasing number of negative regulators that inhibit proinflammatory signal transduction in APCs and other immune cells have been discovered (Tables 1, 2). These negative regulators in APCs function as antigen presentation attenuators (APAs) to control the strength and duration of proinflammatory signaling in APCs in order to set the threshold of antigen presentation (tolerogenic state), regulate the magnitude and duration of antigen presentation, and prevent acute toxic innate immune hyper-response (endotoxin shock) and pathological autoimmunity. Recent studies indicate that cytokine signaling in APCs is critical for antigen presentation (77–80) and is also tightly regulated at multiple points by negative regulators (62, 64).

Additionally, certain subsets of APCs function to hinder immune activation and promote tolerance. Tolerogenic DCs are usually immature DCs, which have low levels of co-stimulatory molecules (75, 81, 82). Immature DCs can induce tolerance through the induction of regulatory T cells that suppress immune responses by secreting anti-inflammatory cytokines such as IL-10 and TGFβ (83). Tolerogenic DCs can also be generated *ex vivo* by treating them with TGF-β or a variety of immunosuppressive drugs. A population of DCs (CD11c^low^, CD45RB^+^) was found to have tolerogenic activity and the ability to induce regulatory T cells in the periphery (84). A recent study showed that plasmacytoid DCs induced the generation of CD25^+^CD4^+^Foxp3^+^ regulatory T cells in lymph nodes (85).

The transcriptional signature of dendritic cells has been investigated to better understand their immune phenotype. A recent study examining the role of RelB, a transcription factor that belongs to the NF-kB/Rel family, in steady-state dendritic cells demonstrated that absence of this transcription factor is associated with increased populations of not only CD25^+^CD4^+^Foxp3^+^ regulatory T cells, but also IL-2-producing CD25^low^CD4^+^CD44^High^ T memory type 1 (Tm1) cells (86). The transcription factor IRF4, on the other hand, has been associated with establishing an immunogenic DC phenotype by promoting Th2 differentiation via IL-10 and IL-33 expression (87), however, further studies have demonstrated that IRF4 is also involved in DC priming of peripheral Foxp3^+^ regulatory T cells (88). Another transcription factor that has been investigated is dendritic cell-specific transcript (DC-SCRIPT), which, when knocked down, promotes expression of IL-10 and decreases expression of IL-12 by DCs (89). To better understand the mechanisms of this, Søndergaard et al. demonstrated that knock down of DC-SCRIPT was associated with decreased expression of MAPK dual-specific phosphatases (DUSP), specifically DUSP4, which concomitantly enhanced ERK signaling, leading to increased IL-10 production (90).

In parallel with classic anti-inflammatory cytokines, other metabolites present in the local immune environment can promote immune tolerance. One critical pathway implicated in generating tolerogenic DCs involves the catabolism of the amino acid tryptophan. DCs can produce indoleamine 2,3 dioxygenase (IDO), the rate-limiting enzyme in the catabolic pathway for tryptophan (91, 92), which degrades the indole moiety of tryptophan, serotonin, and melatonin, and initiates the production of kynurenines. IDO catalyzes the local depletion of the essential amino acid tryptophan, which enhances the production of proapoptotic kynurenines that inhibit T-cell proliferation and promote T-cell apoptosis (91, 92).

Similar to IDO, the enzyme tryptophan-2,3-dioxygenase 2 (TDO) is an enzyme that is involved in the degradation of tryptophan and represents an alternative catabolic pathway. Previously, this enzyme was thought to be expressed only in the liver and neuron, where it is involved in regulating levels of tryptophan systemically and 5-hydroxy-tryptophan in the central nervous system, respectively (93). However, there is increasing evidence suggesting that TDO can be produced by a variety of cancers, including hepatocellular carcinoma, glioma, melanoma, and others (94). In addition, recent studies have demonstrated that certain, specialized myeloid cells can express TDO and contribute to an immunosuppressive microenvironment (95, 96). The exact biologic and molecular basis for such a regulatory myeloid cell subset, however, is unclear.

APC surface ligands, particularly the B7 family molecules, also play an important role in inducing T cell dysfunction to promote peripheral tolerance. At least five B7-family molecules have co-inhibitory function: B7-1 (CD80), B7-2 (CD86), B7-H1 (PD-L1), B7-DC (PD-L2), and B7-H4 (B7x) (97, 98). CD80 and CD86 are the main co-stimulators for T cells through binding to CD28. CD80 and CD86, however, can be co-inhibitory for effector T cells after ligation with CTLA-4. B7-H1 and B7-DC deliver a co-inhibitory signal to effector T cells through ligation of programmed cell death 1 (PD-1). B7-H4 binds to a putative receptor to deliver a negative signal to T cells. Collectively, these B7-family molecules have both co-stimulatory or co-inhibitory activities to balance immune responses (97, 98). In addition to being found on APCs, the B7 family ligands can also be found on peripheral tissue and tumor cells, which we discuss in further detail in the following sections. The APC surface ligands in this family and the T cell receptors they interact with are the main targets of the therapies whose successes have transformed our understanding of cancer medicine and brought about the Era of Checkpoint Blockade Immunotherapy.

### Negative Regulation of T Cells

**Figure 5:**
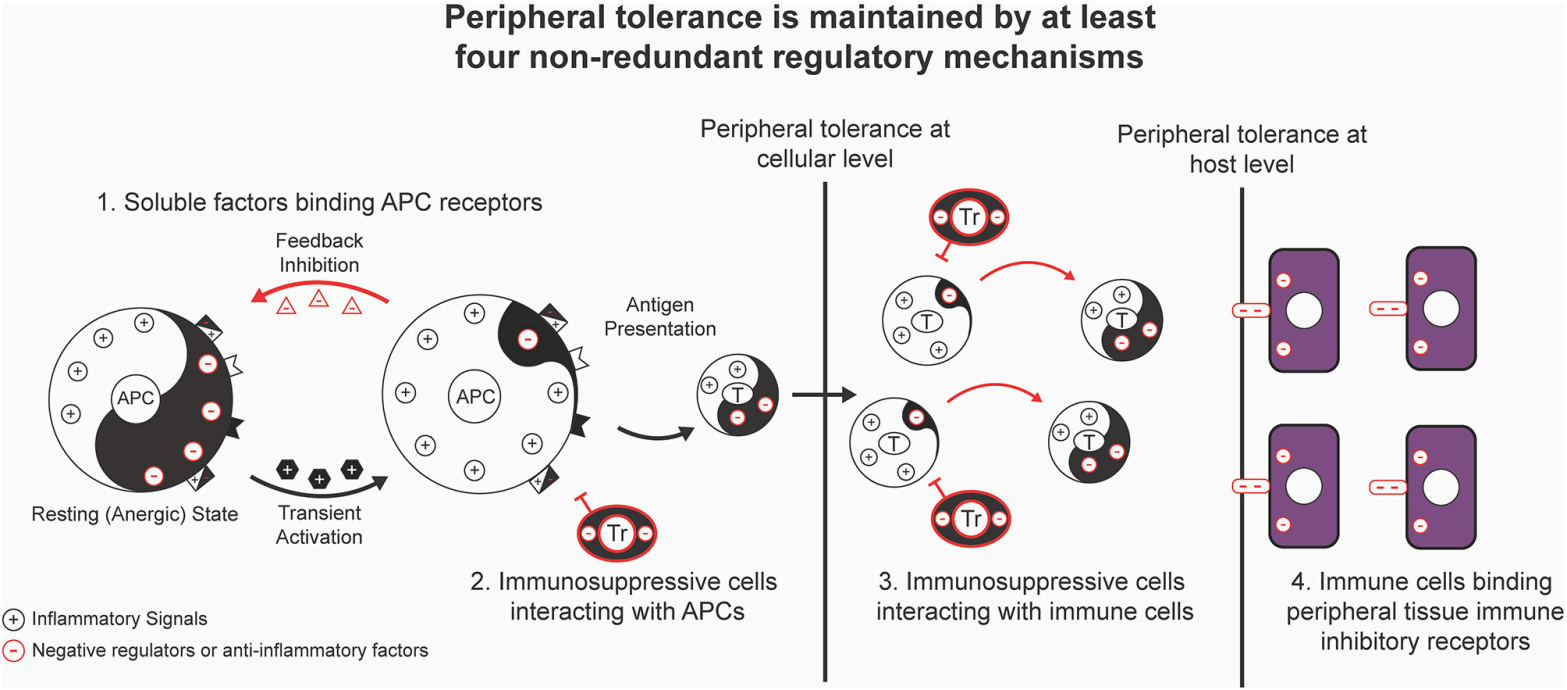
Maintenance of peripheral tolerance at cellular and host levels. Peripheral tolerance is first maintained by individual immune cells using a network of negative regulators [Yin (–)], including negative regulators of inflammatory signaling, soluble anti-inflammatory factors, inhibitory surface receptors and ligands, and regulatory cell subsets, to set up a threshold of stimulatory signaling and keep individual immune cells in an immature or a resting (anergic) state. After activation by proinflammatory stimuli [Yang (+)] that exceed a threshold level, the inducible feedback negative regulators only allow the transduction of transient stimulatory signals, so that temporarily activated immune cells will quickly shift to a post-activation effector state. Peripheral tolerance is also maintained at the host level by an additional network of negative regulators, including inhibitory surface receptors on peripheral tissues and Foxp3^+^ T_reg_ cells. The balanced outcome of the proinflammatory signals mediated by pathogens and negative regulators at both the cellular and host levels results in an appropriate immune response that is sufficient to clear pathogen-infected cells, but insufficient to cause acute innate immune toxicity and autoimmune pathologies to normal cells.

The second level of regulation of peripheral tolerance directly inhibits the activation and effector function of lymphocytes such as T cells. As in the negative regulation of APCs, negative regulators of proinflamamtory signaling, soluble anti-inflammatory factors, inhibitory surface receptors and ligands, and regulatory cell subsets play an important role in the regulation of T cell function (Figures 1, 5). Additionally, negative regulators that control T-cell receptor (TCR) signaling are important in the maintenance of T cell tolerance. In this section, we highlight some negative regulators that act directly on T cells to influence activation and tolerance.

Many of the negative regulators of proinflammatory TLR and cytokine signaling, and anti-inflammatory factors such as IL-10 and TGFβ described above are also important for the regulation of T-cell activation and tolerance (Tables 1, 2). T cells express TLR and cytokine receptors and are subject to the regulation of TLR and cytokine receptor signaling by the same negative regulators described above. T-cell receptor (TCR) signaling is unique to T cells, and activates at least three different families of transcription factors: the nuclear factor of activated T cells (NFAT) family, the activating protein (AP)-1 family, and the nuclear factor NF-κB family (99, 100). The negative regulators of TCR signaling include NFATp, NFAT4, MKP, Cbl, Tob, MKP, Foxj1, Foxo3a, Foxp3, Calcipressins, etc. The detailed roles of many of these regulators of TCR signaling (101), TLR signaling (65), and cytokine receptor signaling (102) in T cells have been recently reviewed.

A regulatory cell subset of T cells, appropriately named regulatory T cells (Tregs), are also important players in the maintenance of peripheral tolerance Figure 5. Forkhead box P3 (Foxp3), a member of the forkhead transcriptional factor family, is a transcription factor that plays an essential role in the development and function of CD4^+^CD25^+^ regulatory T cells (T_reg_) (103, 104). Foxp3^+^CD25^+^CD4^+^ T_reg_ cells play a role in suppressing immune responses in a trans-acting way, mainly *via* the production of anti-inflammatory factors such as IL-10 and TGF-β. Mutations in the gene encoding Foxp3 were identified as the cause of the fatal human autoimmune disorder “immune dysregulation, polyendocrinopathy, enteropathy, X-linked” (IPEX) (21, 22, 105). *Foxp3*-deficient mice or spontaneous *Foxp3* mutant scurfy mice also exhibit severe autoimmune pathologies including dermatitis, lymphoproliferation, and lymphocytic infiltration of multiple organs (23, 24). *Foxp3* is highly expressed in CD25^+^CD4^+^ T_reg_ cells, although low levels of Foxp3 are also expressed in naive and activated CD25^−^CD4^+^ T cells (106). Several lines of evidence indicate that CD4^+^CD25^+^ T_reg_ cell development is critically dependent on Foxp3 expression (103, 104): *Foxp3*-deficient bone marrow cannot give rise to CD4^+^CD25^+^ T_reg_ cells in chimeric wild-type mice; Foxp3 transgene overexpression in mice resulting in an increase in the CD4^+^CD25^+^ T_reg_ cell subset and acquisition of suppressive properties by CD4^+^CD25^−^ and CD8^+^ T cells; the acquisition of regulatory properties by CD4^+^CD25^−^ T cells after retroviral transduction with Foxp3; and the association of induced Foxp3 expression with acquired regulatory functions of non-regulatory T cells in both humans and mice.

Like other forkhead transcriptional factors (107), Foxp3 binds DNA and acts as a transcriptional activator or repressor. Foxp3 may function as a transcriptional repressor of proinflammatory cytokine genes, because Foxp3 can bind to consensus forkhead binding domains adjacent to NFAT transcription factor binding sites in the promoters of several cytokine genes, such as IL-2, IL-4, and TNF (108). Foxp3-targeted genes and transcriptional regulation, however, are still not defined. Collectively, Foxp3 functions as a negative regulator of immune responses by repressing the production of proinflammatory cytokines in a variety of cell types, including CD25^+^CD4^+^ T_reg_ cells, which, together with other regulatory cells (75, 109), provide one of many non-redundant regulatory mechanisms for maintenance of peripheral tolerance (Figure 5).

There additionally exist populations of regulatory CD4^+^ T cells that lack expression of Foxp3, such as Tr1 cells that are uniquely identified as expressing both cell-surface markers CD49 and LAG-3, and produce high levels of IL-10 and TGF-β (110, 111). In addition to producing anti-inflammatory cytokines, Tr1 cells are capable directly lysing myeloid APCs by secreting granzymes and perforins via a mechanism dependent on HLA class 1 recognition, CD54/LFA-1 adhesion, killer cell Ig-like receptors (KIRs), CD2, and CD226 on Tr1 cells (112). Recent studies have elucidated the differentiation pathway for Tr1 cells–IRF1 and BATF have been implicated as pioneer factors for Tr1 cell development in response to IL-27 signaling (113). Additionally, IL-2 inducible T cell kinase (ITK), serves an important role as ITK deficiency lead to diminished expression of AHR, cMAF, and IRF4, transcription factors involved in Tr1 cell development (114); IRF4 in particular can modulate expression of Blimp-1, and together, induce IL-10 production (115).

In addition to regulatory CD4^+^ T cells, recent studies have identified regulatory CD8^+^ T cells that suppress naïve T cell proliferation and are vital for maintaining self-tolerance (116–120). Compared to other CD8^+^ T cells, regulatory CD8^+^ T cells express higher levels of markers such as CTLA-4 and ICOS, and constitutively express CD25, increasing their sensitivity to IL-2 (116). When examining the transcriptional profile, regulatory CD8^+^ T cells more closely resemble regulatory CD4^+^ T cells compared to other, canonical CD8^+^ T cells (121). One pathway implicated in the differentiation and function of CD8^+^Foxp3^+^ T cells is TNF/TNFR2, and TNFR2 may be used as a marker to identify these cells (122, 123). A key mechanism by which regulatory CD8^+^ T cells can mediate naïve CD4^+^ T cell proliferation is via TGF-beta and IFN-gamma secretion (124); TGF-beta is also partially involved in promoting regulatory CD8^+^ T cell differentiation via p38 MAPK signaling (125). A different mechanism to mitigate CD4^+^ T cell proliferation utilized by subsets of regulatory CD8^+^ T cells that express high levels of CD11c employs the Fas-FasL pathway in an antigen-independent manner to mediate direct cytotoxicity (126). It is becoming more appreciated that both CD4^+^ and CD8^+^ regulatory T cells strongly contribute to maintaining immune tolerance, and may serve to increase an individual's immune threshold.

A regulatory subset of B cells, termed regulatory B (Breg) cells have been gaining recognition as playing in important role in suppressing the immune response by producing cytokines such as IL-10, IL-35, and TGF-β (127–129). Breg cells were initially postulated to exist and exert an immunosuppressive effect upon the observation that B cell deficient mice had more severe disease and delayed recovery in a murine model of experimental autoimmune encephalomyelitis (130). There exist multiple subsets of Breg cells, including B10 cells, regulatory plasma cells, Marginal Zone (MZ) B cells, and many others, and the majority of these subsets can be identified by high surface expression of CD1d (131). CD1d belongs to the CD1 family of cell surface receptors that resemble MHC I, collectively bind lipid molecules by their hydrophobic carbon chain, and presents these antigens to invariant natural killer T (iNKT) cells (132, 133). One such molecule that is bound by CD1d on B cells is alpha-galactosylceramide, a potent iNKT cell agonist that performs a vital role in regulating immune tolerance (133, 134). Mechanistic studies have revealed that lipid presentation on CD1d by Breg cells drives differentiation of IFN-gamma^+^ iNKT cells, which successively can inhibit Th1 and Th17 responses (135).

One of the key cytokines implicated in driving Breg cell function is IL-10, and this large subset of IL-10-producing Breg cells is known collectively as B10 cells, the most extensively characterized of Breg cells (136, 137). Yanaba et al. utilized high cell surface expression of CD1d and CD5 to identify B10 cells, however, there is no consensus set of cell surface markers to uniformly define this subset of Breg cells (138, 139). While the precise molecular mechanism inducing B10 cell differentiation is unclear, interactions with T cells do not seem to be a necessary prerequisite, as nude mice lacking T cells can still possess B10 cells (139). B10 cells have been shown to have the ability to regulate antigen-specific immune responses in the presence of IL-21 and CD40-based interactions with T cells (140). In a murine model for collagen-induced arthritis, B10 cells suppressed Th1 and Th17 differentiation in an IL-10-dependent fashion as mice with IL-10^−/−^ B cells developed more severe disease, increased populations of Th1 and Th17 cells, and decreased populations of Tr1 cells compared to mice with wild-type B cells (141). While B10 cells secrete IL-10 to dampen the immune response, following *il10* transcription, the genes encoding transcription factors Blimp-1 and IRF4 are transcribed, indicating an ability to further differentiate into plasma cells, possibly to promote clearance of antigens via antibody-mediated processes (142). In humans, there exists a rare population of CD19^+^CD24^hi^CD38^hi^ B cells that that serve to regulate the immune response partially via IL-10 (143). Concordantly, in patients with rheumatoid arthritis, CD19^+^CD24^hi^CD38^hi^ B cells are not only presents in reduced numbers, but also have a decreased ability to suppress naïve T cell differentiation into Th1 and Th17 cells and promote differentiation into T regulatory cells (144).

In addition to IL-10, IL-35, which amongst B cells is produced primarily by CD138^+^ plasma cells, has been increasing investigated in its immune regulatory function, which parallels those of IL-10 (129, 145, 146). IL-35 belongs to the IL-12 family of cytokines and is comprised of p35, the IL-12-alpha chain, and Epstein-Barr virus-induced gene 3 (Ebi3) (147). IL-35 itself is partially governed by a positive feedback loop as IL-35 signaling enhances binding of STAT1 to p35 and Ebi3 promotors in a subset of Foxp3^−^ regulatory T cells known as “iTR35” cells, and successively increases expression and secretion of these proteins (148). Decreased expression of IL-35 has been reported in multiple autoimmune disease states, including inflammatory bowel disease, Sjogren syndrome, type 1 diabetes, and others (148–150). Importantly, treatment of mice with IL-35 strongly promoted expansion of IL-10-producing Breg cells and decreased IL-17 expression (151, 152). Furthermore, IL-35 has been shown to modulate activation and antigen presenting capabilities of B cells, as mice with p35^−/−^ B cells expressed higher levels of activation markers such as CD44 and CD69, and molecules involved in antigen presentation such as MHC-II, CD80, and CD86 (129). While further studies detailing the molecular mechanisms governing Breg cell interactions with T cells and other immune cells, it is becoming increasing clear that Breg cells serve an important role in regulating the immune response.

### Negative Regulation in Peripheral Tissues

The third level of regulation of peripheral tolerance occurs in peripheral, non-lymphoid tissues. Various regulatory mechanisms (Figure 1) are used by peripheral tissues to protect themselves against self-reactive T cells. The defense mechanisms of peripheral tissues are important for the maintenance of peripheral tolerance at the host level, when other regulatory mechanisms are unable to restrict the activation of pathogenic self-reactive T cells at the cellular level.

The negative regulators of proinflammatory signaling are also expressed in peripheral tissues and play a role in maintaining peripheral tolerance at the host level. For example, it was reported that diabetes-prone NOD mice harboring beta-cells expressing a SOCS1 transgene had a markedly reduced incidence of diabetes, and the disease protection was correlated with enhanced suppression of STAT1 phosphorylation in SOCS1-expressing beta-cells (153).

Peripheral tolerance also utilizes the Fas ligand **(**FasL) response pathway to regulate apoptosis of self-reactive T cells. FasL (CD95L, Apo-1L, CD178), a member of TNF family, binds to its cognate surface receptor Fas (CD95, Apo-1) and triggers the extrinsic apoptotic pathway, leading to the death of target cells (154). The Fas/FasL apoptotic pathway is highly regulated, and its abnormal regulation has been associated with autoimmunity and cancer. Although predominantly expressed on activated T cells and NK cells, FasL is also expressed on non-hematopoietic cells such as brain, lung, and immunologically privileged sites to protect these tissues and organs from pathogenic self-reactive T cells (155).

As seen at the levels of APCs and lymphocytes, B7 family members also play a critical role in maintaining self-tolerance at the level of peripheral tissues. PD-L1, B7-H3, and B7-H4, are important for protecting peripheral tissues against pathogenic self-reactive T cells (98, 156). A hint of the protective role of B7 molecules comes from the observation that inhibitory B7 members such as PD-L1 and B7-H4 are also expressed on non-lymphoid tissues such as heart, muscle, lung, kidney, liver, and various cancer cells. Recently, Keir et al. experimentally demonstrated that PD-L1 expressed on pancreatic islets prevented diabetes by providing an inhibitory signal to infiltrating self-reactive CD4^+^ T cells (156). Chronic stimulation of T cells by these inhibitory B7 family ligands can lead to a semi-permanent downregulation of T cell function. Tumors exploit this system, overexpressing these ligands to create a tolerogenic microenvironment that fosters tumorigenesis.

The negative regulatory mechanisms employed by peripheral tissues serve as a last line of defense against the development of autoimmunity. Taken together with the negative regulatory mechanisms employed by APCs, T cells, and B cells, this coordinated system maintains a heightened threshold for immune activation. Therefore, the ability of cancer cells, which originate from normal tissue, to utilize and upregulate these same mechanisms creates an critical barrier to generating an effective anti-tumor immune response.

## T Cell Exhaustion in Cancer

While peripheral tolerance acts to delete antigen specific T cells as a natural means of protection, T cell exhaustion, described more than a decade ago, is a state of T cell differentiation that becomes evident during persistent T cell stimulation. T cell exhaustion has been described in chronic viral, bacterial, and parasitic infections and in cancer in various animal models and humans, and the functional depiction continues to become clearer. T cell exhaustion can be best understood by considering the extrinsic and cell-intrinsic pathways responsible for negative regulation of T cell function.

### T Cell Response to Antigen and Loss of Effector Function

Upon acute antigen exposure, naïve CD8^+^ T cells expand and differentiate into cytotoxic effector cells to control and clear the antigen. Further differentiation results in either functional T cell memory or T cell dysfunction as in self-tolerance or exhaustion. The fate of the naïve T cell is dependent on the context of antigen stimulation and defined by unique genetic signatures that determine the functional and phenotypic properties of each T cell differentiation state. (157, 158). When self-reactive CD8^+^ T cells come in contact with self-antigen in the tolerogenic setting of the peripheral tissues, they adopt a state of unresponsiveness. Tolerant T cells can become activated by various conditions that promote T cell proliferation (including cytokines IL-2 and IL-15, and lymphopenia), but this rescue of T cell function is transient, and self-reactive T cells are re-tolerized in the absence of proliferative stimulation.

Another state of T cell dysfunction is T cell exhaustion. Rather than a state of irreversible, terminal differentiation or functional unresponsiveness, T cell exhaustion is characterized by an adaptive state of hyporesponsiveness. By maintaining a low level of cytotoxic function, exhausted T cells are able to help control a chronic threat without producing a full blown immune response that could potentially induce fatal disease in the host (159). Properties of T cell exhaustion were first identified in lymphocytic choriomeningitis virus (LCMV) infection, where chronic infection led to persistent antigen presentation (160). In chronic LCMV infection, virus-specific CD8^+^ T cells that do not produce typical cytotoxic molecules were identified, suggesting that highly cytotoxic T cells generated during initial responses are lost upon prolonged antigen presentation. Soon after, studies described that during exhaustion, loss of function occurs in a hierarchical manner, where IL-2 production and proliferative capacity are lost first, followed by loss of tumor necrosis factor (TNF) production at intermediate stages of dysfunction. Finally, severe exhaustion can lead to complete loss of the ability to produce IFNγ, and the final stage of exhaustion is followed by physical deletion of antigen-specific T cells.

A key property of memory CD8^+^ T cells is the ability to survive without antigen stimulation via interleukin (IL)-7 and IL-15 to mediate self-renewal. This feature is lost upon exhaustion, as they have decreased expression of CD122 (the B-chain of the IL-2 and IL-15 receptor) and CD127 (the IL-7 receptor a-chain). As a result, these T cells switch to using their cognate antigen and epitope-specific TCR signals for long-term maintenance. Studies in mice have revealed that when exhausted T cells are adoptively transferred into antigen-free recipients, there is a disappearance of exhausted cells but also minimal recovery of memory CD8^+^ T cells. This verified that once T cells are committed to exhaustion, antigen removal does not restart the memory T cell differentiation process (161). Epigenetic studies have revealed that exhausted CD8^+^ T cells have an accessible chromatin landscape, and corresponding program of genes, that is distinct from memory T cells (162). Specifically, a seminal study by Sen et al. identified a region in the *Pdcd1* gene locus demonstrated significant chromatin accessibility only in exhausted CD8^+^ T cells (162). Mechanistically, *de novo* DNA methylation is critical to differentiation into an exhausted subtype, and these DNA methylation programs can be acquired by tumor-infiltrating CD8^+^ T cells (163). Importantly, administration of DNA demethylating agents prior to CBI may avoid T cell exhaustion in tumor-infiltrating CD8^+^ T cells (163). Exhausted T cells, however, can be rescued, and this principle has had significant clinical implications on cancer immunotherapy, which we discuss later in this review.

### Negative Regulatory Pathways Leading to Exhaustion

Functional, phenotypic, and molecular analyses have revealed that despite overlapping traits, many of the states labeled in the literature as “anergy” are regulated and maintained by distinct factors, and require different strategies to restore cell function. While there are various states of T cell dysfunction, T cell exhaustion is a progressive, long-term process which involves various negative regulatory mechanisms. These mechanisms can be grouped into three main categories: (1) cell surface inhibitory receptors, (2) soluble factors, and (3) immunoregulatory cell types. These specific negative regulatory mechanisms distinguish exhausted T cells from anergic, tolerant, or ignorant T cells.

Inhibitory receptors on the surface of T cells play vital roles in many aspects of self-tolerance and prevention of autoimmunity. While T cells transiently express inhibitory receptors upon activation, prolonged and/or high expression of multiple inhibitory receptors is a defining feature of exhaustion (164).

PD-1 is expressed on antigen-activated T cells and upregulated in T cell exhaustion (165). In the presence of its ligands, PD-L1 and PD-L2, on the surface of APCs and normal and cancerous peripheral tissue, PD-1 functions to suppress T cell inflammatory activity. Binding of either of these ligands to PD-1 results in the recruitment of phosphatases SHP-1 and SHP-2 to the phosphorylated cytoplasmic ITSM domain of the receptor. These phosphatases then counteract kinases important for signal transduction downstream of TCR, CD28, and other costimulatory receptors. By inhibiting activation of the PI3K and Akt pathways among others, PD-1 signaling results in decreased proliferation, IL-2 production, protein synthesis, and survival of T cells (166). PD-L1 is expressed on various cells throughout the body, protecting tissues from immune-mediated damage (156). Like CTLA-4, PD-1, as well as PD-L1, are highly expressed on T_regs_, and activation of the PD-1/PD-L pathway favors the differentiation of naïve T cells into Tregs to create a more immune-suppressive environment.

In addition to PD-1, various other inhibitory surface receptors are important in regulatory T cell exhaustion. As discussed above, CTLA-4 shares ligands, B7-1 (CD80) and B7-2 (CD86), with CD28, a co-stimulatory receptor required for the second signal in T cell activation. CTLA-4 binds B7-1 (CD80) and B7-2 (CD86) with 10-20 times greater affinity than CD28, resulting in competitive inhibition of the co-stimulatory receptor. CTLA-4 binding also leads to endocytosis of B7 molecules, decreasing the availability of these ligands on APCs, peripheral tissue, and tumor cells for T cell co-stimulation via CD28.

CTLA-4 is an important negative regulator at the level of lymphocytes, and functions to modulate the extent of early T cell activation via two main mechanisms. First, upon activation, T cells start expressing CTLA-4, which blocks further activation and blunts the immune response. As a target gene of FOXP3, the transcription factor whose presence defines the T_reg_ cell lineage, CTLA-4 is also expressed constitutively in high levels on T_regs_, enabling them to prevent the activation of other T cells (24, 167, 168). CTLA-4 acts on two major subsets of CD4^+^ T cells, dampening helper T cell activity while boosting the immunosuppressive functions of T_regs_. CTLA-4 also has some direct effect on CD8^+^ T cells, negatively regulating their proliferation and activation (169).

Two additional inhibitory receptors are also important in T cell exhaustion: lymphocyte activation gene-3 (LAG3) and T cell immunogloblulin and mucin domain 3 (TIM3). LAG3 was discovered over 25 years ago as a receptor that is upregulated on activated CD4^+^ and CD8^+^ T cells and also natural killer (NK) cells, and are detectable on a T cell as early as 24 h post activation (170). LAG3 structurally resembles the CD4^+^ receptor and binds to MHC Class II, but its functional impact on CD8 and NK cells, which only interact with MHCI, implies that LAG3 also has alternative ligands. Liver sinusoidal endothelial cell lectin (LSECtin) and Galectin-3 have been speculated to be additional ligands for LAG3 and have been found to be expressed on cancer cells to inhibit IFNγ secretion by CD8^+^ T cells (171). Recently, fibrinogen-like protein 1 (FGL1) has been identified as a high-affinity LAG3 ligand that can inhibit antigen-specific T cell responses and is upregulated in various cancers such as lung adenocarcinoma, prostate cancer, and breast cancer (172).Moreover, LAG3 has also been found on T_reg_ cells, and blocking LAG3 diminishes suppressor function (173). TIM3 has been shown to play an inhibitory role in T cell immune responses. TIM3 is expressed on CD4^+^ and CD8^+^ T cells and is correlated with reduced amounts of cytokine production and also decreased proliferation, suggesting that they play a role in exhausted T cells. T cells isolated from chronic viral infections and various human cancer samples include a fraction of antigen-specific, nonfunctional CD8^+^ T cells that coexpress LAG3/TIM3 and PD-1 (160). In these models, blockade of both receptors resulted in an improved and synergistic antiviral and anti-tumor immune response compared to singular blockade (174, 175).

### Transcriptional Definitions of Exhaustion

Though exhausted cells share similar phenotypes, different stimuli or stimuli from different species may generate different molecular and transcriptional profiles that further distinguish them. Genomic approaches can provide a more detailed landscape of exhausted T cells to provide us a fuller understanding. For example, global transcriptional profiling has shown that exhausted CD8^+^ T cells are distinct from effector and memory T cells in terms of TCR and cytokine signaling pathways, migratory potential, chemokine expression, and metabolism (176). Genomic profiling of exhausted cells is important as it can potentially determine whether exhaustion is a fixed lineage or if plasticity exists for them to become fully functional effector or memory.

Several transcriptional pathways have already been identified for T cell exhaustion. B lymphocyte-induced maturation protein-1 (Blimp-1). Blimp-1 is a transcriptional repressor and is a master regulator of terminal B cell differentiation (177). Kallies et al. however, has recently reported that Blimp-1, found on CD4^+^ and CD8^+^ T cells, is also a negative regulator of T-cell differentiation and function. *Blimp-1*-deficient mice die during late gestation, and *Rag1*^−/−^ mice reconstituted with fetal liver stem cells from *Blimp-1*-deficient mice show severe inflammation and multiorgan autoimmune disease. Moreover, Blimp-1 is expressed significantly higher in exhausted T cells relative to effector T cells, and is associated with the upregulation of many inhibitory receptors, including PD-1, LAG-3, CD160, and CD244. Ablation of *Blimp-1* reverses expression of these receptors and restores memory differentiation, suggesting that lesser activity of Blimp-1 promotes formation of memory cells, intermediate amounts promote terminal differentiation of effector activity, and higher amounts generate exhaustion (178). Moreover, another transcription factor, T-bet, has a parallel role to Blimp-1 in mediating terminal differentiation of CD8^+^ T cells after clearance of antigen. T-bet promotes sustained responses during chronic viral infection and represses transcription of inhibitory receptors. Thus, Blimp-1 and T-bet represent the main transcriptional regulation nodes involved in the exhaustion of CD8^+^ T cells.

T-bet has also been shown to have a cooperative relationship with another transcription factor, Eomesodermin (Eomes). During early stages of CD8^+^ T cell activation, T-bet and Eomes cooperate to bring out cytotoxic function by inducing expression of perforin and granzymes (179). In addition, Eomes has been largely implicated in driving memory differentiation, mainly through promoting IL-15 signaling (180). While there is expressional overlap between the two, their functional roles are not necessarily reciprocal. T-bet represses expression of inhibitory receptors by direct binding to the promoter region of PD-1, while Eomes is associated with expression of numerous inhibitory receptors. Lastly, T cells with high levels of Eomes and PD-1 exhibited higher Blimp-1, inhibitory receptors, and are associated with a severe state of exhaustion (181). Importantly the threshold model proposed here relates primarily to the initial activation of endogenous antigen specific immune responses. After the threshold is crossed and adaptive immune responses engage a pathogen then a natural and physiologic contraction phase eventually occurs in attempt to return back to homeostasis or new baseline. This contraction phase occurs irrespective of whether a pathogen or tumor is successful eradicated or not and may involve memory formation in both cases or T-cell exhaustion in the later. Eomes plays a key role during this contraction phase and is thus involved in both memory formation and T-cell exhaustion. Indeed, negative regulators such as Eomes and even PD-1 have a physiologic role as negative regulation is critical for maintaining homeostasis as well as memory formation during an immune response.

Transcriptional profiling also indicates that the nuclear factor of activated T cells (NFAT) transcription factor is highly expressed in exhausted CD8^+^ T cells. The NFAT family, NFAT1, NFAT2, and NFAT4, are transcription factors that promote expression of a panel of genes required for T cell activation and induction of numerous effector cytokines. However, recent studies have shown that they have an additional role that serves to limit the immune response. The duality of NFAT function allows these transcription factors to participate in multiple programs regulating different cell types in different signaling contexts. Importantly, the link between NFAT and T cell tolerance and anergy makes it a favorable target for cancer. Lower NFAT function and dysregulation is associated with poor cytokine production. In addition, NFAT can also regulate PD-1 expression after *in vitro* activation of T cells, but the link between nuclear translocation of NFAT and PD-1 expression has yet to be determined.

## Overcoming Tumor-Induced T Cell Dysfunction

### Tumors Hijack Host Negative Regulatory Mechanisms

In addition to the barrier of peripheral tolerance at the host level, tumors develop strategies to hijack the host's negative regulatory mechanisms in order to sabotage antitumor immunity, raising the threshold for anti-cancer immunity. Tumors can produce soluble anti-inflammatory factors such as IL-10, TGFβ, and others (182, 183), and express inhibitory surface receptors such as B7-H4, PD-L1, and FasL (184, 185). Cancer-associated viruses such as human papillomavirus (HPV) can interrupt immune responses by up-regulating the PI3-K pathway and down-regulating MAPK pathways (186). The enhanced expression of various natural negative regulators by tumor cells establishes an immune-suppressive stromal microenvironment (187), further dampening antitumor responses. In addition, Foxp3^+^ T_reg_ cells are attracted to tumors, where they inhibit the function of infiltrating immune cells (184).

### Inhibiting Negative Regulators to Enhance Anti-tumor Immunity

Inhibition of negative regulators that are critical for maintaining peripheral tolerance may overcome both peripheral tolerance at the host level and tumor-mediated immunosuppression, since tumors do not “invent” unique immunosuppression mechanisms, but utilize and hijack the host's natural negative regulatory mechanisms, likely via mutations and selective pressure (188). By inhibiting a key negative regulator, a patient's immune threshold can be effectively lowered, decreasing the magnitude of proinflammatory stimulation required to induce an immune response.

### Checkpoint Blockade Immunotherapy

Checkpoint blockade immunotherapy (CBI) is one of the most promising examples of the application of this principle in the treatment of cancer. The term “immune checkpoint” is occasionally used as an umbrella term to refer to any of the immune-inhibitory pathways. To maintain consistency with the definition of checkpoint blockade immunotherapy as therapeutic agents that interfere with inhibitory T cell surface receptor-ligand engagement, we will consider only inhibitory T cell surface receptors and their downstream immune-inhibitory pathways to be immune checkpoints.

The multitude of inhibitory pathways that play a critical role in maintaining self-tolerance and regulating the duration and amplitude of the immune response are called immune checkpoints.

Tumors have been shown to adapt and upregulate many these immune-inhibitory pathways to evade immune detection and destruction. An important example of this is the increased expression of the inhibitory ligands that modulate T-effector functions, which has been observed in various types of cancers.

In contrast with most antibodies currently used in cancer therapy which target tumor cells, antibodies for immune checkpoint blockade target lymphocyte receptors and their ligands which are present on the surface of APCs as well as cancerous and normal cells in peripheral tissues. When these antibodies bind their targets, they prevent the normal ligand-receptor interaction that would initiate an important immune-inhibitory pathway. By knocking out a critical pathway used by tumor cells to evade the immune system, these therapies effectively release the brakes on the immune system, lowering the immune threshold and enabling the development of an anti-tumor immune response. Antibodies developed to inhibit CTLA4 and PD-1 are revolutionizing cancer immunotherapy and have brought us into the era of Checkpoint Blockade Immunotherapy.

CTLA-4 was the first immune-checkpoint antigen to be targeted in CBI, and clinical testing of ipilimumab and tremelimumab, two humanized CTLA-4 antibodies, began in 2000 (70). Ipilimumab became FDA approved for the treatment of advanced melanoma in 2011, and treatment with this drug showed incredible results (189). In a landmark phase 3 study on Ipilimumab for the treatment of advanced melanoma by Hodi et al. Ipilimumab alone demonstrated a disease control rate of 28.5%, and 1 and 2-year survival rates of 45.6 and 23.5%, respectively (189). The impressive effects on long-term survival also supported the idea that immunotherapies could potentially re-educate the immune system to induce anti-tumor responses that are sustained long after completion of therapy. By blocking CTLA-4 signaling the level of co-stimulation required to activate the CD28 pro-immunogenic pathway is decreased, thus directly reducing the threshold for activation at this step. The early success of CTLA-4 blockade revealed the potential of CBI and how it could change the meaning of being diagnosed with advanced melanoma.

Antibodies that target the PD-1/PD-L1 pathway have also significantly improved patient outcomes in multiple clinical studies (69). Analogously to anti-CTLA-4, by blocking PD-1 signaling the existing co-stimulatory factors and TCR signaling can more readily activate T-cells, thus directly reducing the threshold for activation at this step. Two such antibodies that have been extensively studied are pembrolizumab and nivolumab. The KEYNOTE-001 phase I study of 173 patients treated with pembrolizumab for advanced or unresectable melanoma that had progressed on ipilimumab and a BRAF inhibitor showed an overall response rate (ORR) of 26%(190). This study lead to FDA approval of the first anti-PD-1 antibody, pembrolizumab, for advanced or unresectable melanoma. Several months later, the results of the CheckMate 037 trial supported FDA approval of nivolumab as second-line therapy for unresectable or metastatic melanoma (191). The phase II and III trials of PD-1 inhibitors in melanoma that followed, including KEYNOTE-006, KEYNOTE-002, CheckMate 037, CheckMate 069, and CheckMate 067, provided the basis to expand FDA approval of these PD-1 inhibitors as first-line therapy for untreated advanced melanoma regardless of *BRAF* mutation status, for ipilimumab-refractory melanoma, and in combination with ipilimumab as first-line therapy for unresectable or metastatic melanoma (nivolumab) (71, 192, 193).

These studies in melanoma served as the impetus for expanding the clinical application of PD-1 inhibitors and exploring CBI in various tumor types. Further studies lead to FDA approval of pembrolizumab for non-small cell lung cancer (NSCLC) in 2015, head and neck squamous cell carcinoma (HNSCC) in 2016, Hodgkin lymphoma, urothelial carcinoma, all tumors with high microsatellite instability (MSI-high), and gastric cancer in 2017. Nivolumab also received FDA approval for treatment of renal cell carcinoma (RCC) in 2015, Hodgkin lymphoma and HNSCC in 2016, and urothelial, MSI-high colorectal, and hepatocellular caricinoma (HCC) in 2017 (194).

The efficacy of anti-PD-L1 antibodies, which inhibit the PD-1/ PD-L1 axis from a different angle, is also being studied. Three PD-L1 inhibitors, atezolizumab, avelumab, and durvalumab, have been clinically tested. Atezolizumab was approved as second-line therapy for metastatic NSCLC in 2016 after the results of the POPLAR and OAK trials (195, 196). A phase II trial studying atezolizumab for urothelial cancer (UC), IMVigo 210, showed an overall response rate (ORR) of 15% in patients with advanced and metastatic platinum-based chemotherapy-refractory UC, a significant improvement over the historical 10% response rate. (197). A separate cohort of the IMVigor 210 trial showed similar results in patients with treatment-naïve metastatic UC, leading to the accelerated approval of atezolizumab as first-line therapy for cisplatin-ineligible advanced and metastatic UC (198). Avelumab and durvalumab, two other anti-PD-L1 antibodies, were approved for merkel cell carcinoma (MCC) and UC, and UC, respectively in 2017 (199–201). These drugs, as well as the other PD-1 and PD-L1 inhibitors, are continuing to be studied in the clinic in various tumor types and in combination with other cancer therapies.

## Autoimmunity and Immune Related Adverse Events (irAEs)

The use of cancer immunotherapies has led to the recognition and characterization of a new category of side effects, immune-related adverse events (irAEs). The irAEs of CBI are relatively mild and rare compared to those seen in other systemic cancer immunotherapies. Of the CBIs, anti-CTLA-4 is associated with the most frequent and severe irAEs. Severe drug-related irAEs were seen in 15–30% of patients on anti-CTLA-4 therapy, sometimes even resulting in fatalities (202). These irAEs included inflammation of normal tissues such as the gut, skin, and endocrine glands. The occurrence of irAEs in individuals with no previous history of autoimmunity reflects the ability of CBI to decrease the threshold of immune stimulation required to elicit an immune response. In contrast to CTLA-4, PD-1 blockade is thought to act primarily within the tumor microenvironment and has been associated with fewer and less severe irAEs in the clinic.

Differences in response to anti-CTLA-4 therapy or anti-PD-1 therapy are reflected in knockout mouse models. Mice deficient in *CTLA-4* die from lymphoproliferation, while mice lacking *PD-1* have more model-dependent autoimmunity, such as arthritis and cardiomyopathy. These findings translate to differences in irAE severity in the clinic. PD-1 was first suspected of playing a role in the regulation of T-cell tolerance and autoimmunity when Nishimura et al. observed that PD-1 knockout mice developed mild glomerulonephritis and detectable autoantibodies, mimicking late onset lupus-like disease (203). Organ-specific toxic effects are observed in patients treated with anti-PD-1 and anti-CTLA-4. Colitis and hypophysitis are more common with anti-CTLA-4 therapy, while pneumonitis and thyroiditis appear to be more common with anti-PD-1 therapy. This suggests that these therapies throw off the negative regulation in peripheral tissues. irAEs can act as a gauge to measure how therapy is shifting the balance between inflammation and tolerance.

Whether the severity of autoimmune side effects correlates with efficacy of CBI induced antitumor responses is an important question. Several studies have shown that patients with more irAEs also have higher response rates. A recent retrospective study of 134 patients with NSCLC treated with nivolumab (anti-PD-1 antibody) found that the development of irAEs was associated with increased overall survival (204). Still, it is not clear what causes the variation in irAEs between patients. It is possible that germline genetic factors and gastrointestinal microbiologic composition can affect baseline host immunity. Certain genes have been shown to increase the risk of autoimmune diseases and studies have just begun to investigate whether these genetic factors increase the risk of irAEs. In addition, it has been demonstrated that presence of bacteria from the Bacteroidetes phylum is correlated with reduced rates of ipilimumab-induced colitis. It may be possible to skew the immune response toward anti-cancer effector functions by increasing the cancer antigen load via combinatorial treatments with chemotherapy and radiation therapy, however, how to achieve only an anti-cancer immune response while completely avoiding irAEs is not yet clear. A multitude of factors can influence the presence and severity of irAEs, therefore, the general consensus is that irAEs are not a necessary outcome of an efficient CBI-induced antitumor response, but that their presence may be indicative of better response to CBI in some patients.

## Conclusion

In conclusion, with the success of checkpoint blockade immunotherapy we now have the capability to robustly activate the immune system and break self-tolerance to induce anti-tumor immunity and/or auto-immunity. Each individual likely has a baseline threshold for immune activation against self and foreign antigens which is a function of multiple complex and interdependent regulatory mechanisms. A better understanding of these immuno-regulatory pathways and activation thresholds is needed to guide rationale and strategic use of combinatorial therapies that enhance anti-tumor immune responses while limiting immune related toxicity. The threshold model we describe here provides a conceptual framework for understanding activation of immunity, tumor responses, and toxicity in the era of checkpoint blockade immunotherapy.

## Author Contributions

KG, VW, S-YC, and AS performed the scientific literature search, wrote the review, and interpreted the data. KG, S-YC, and AS produced the figures. KG, SK, VW, SP, EC, S-YC, PS, SS, and AS provided critical review of the manuscript and approved the final manuscript.

### Conflict of Interest Statement

SP receives research funding from: MedImmune, Genentech, Pfizer, Amgen, Xcovery, Lilly, Bristol-Myers Squibb, and he receives speaking fees from: Boehringer Ingelheim, Merck. EC reports research funding from Pfizer, Merck, AstraZeneca, and Bristol-Myers Squibb outside the submitted work. AS reports research funding and honoraria from Pfizer and Varian Medical Systems, consultant fees from Astrazeneca, and other fees from Raysearch and Merck. The funder played no role in the study design, the collection, analysis or interpretation of data, the writing of this paper or the decision to submit it for publication. The remaining authors declare that the research was conducted in the absence of any commercial or financial relationships that could be construed as a potential conflict of interest.
